# The evolution of different maternal investment strategies in two closely related desert vertebrates

**DOI:** 10.1002/ece3.2838

**Published:** 2017-03-31

**Authors:** Joshua R. Ennen, Jeffrey E. Lovich, Roy C. Averill‐Murray, Charles B. Yackulic, Mickey Agha, Caleb Loughran, Laura Tennant, Barry Sinervo

**Affiliations:** ^1^ Tennessee Aquarium Conservation InstituteChattanoogaTNUSA; ^2^ U.S. Geological SurveySouthwest Biological Science CenterFlagstaffAZUSA; ^3^ Nongame BranchArizona Game and Fish DepartmentPhoenixAZUSA; ^4^ Department of Wildlife, Fish, and Conservation BiologyUniversity of California, DavisDavisCAUSA; ^5^ Department of BiologyUniversity of New MexicoAlbuquerqueNMUSA; ^6^ Department of Ecology and Evolutionary BiologyUniversity of California at Santa CruzSanta CruzCAUSA; ^7^ Present address: Desert Tortoise Recovery OfficeUnited States Fish and Wildlife ServiceRenoNV89502USA

**Keywords:** benign environment hypothesis, bet‐hedging, environmental predictability, offspring fitness, optimal egg size, phenotypic diversification, reproductive trade‐off, sexually antagonistic selection, tortoise

## Abstract

We compared egg size phenotypes and tested several predictions from the optimal egg size (OES) and bet‐hedging theories in two North American desert‐dwelling sister tortoise taxa, *Gopherus agassizii* and *G. morafkai*, that inhabit different climate spaces: relatively unpredictable and more predictable climate spaces, respectively. Observed patterns in both species differed from the predictions of OES in several ways. Mean egg size increased with maternal body size in both species. Mean egg size was inversely related to clutch order in *G. agassizii*, a strategy more consistent with the within‐generation hypothesis arising out of bet‐hedging theory or a constraint in egg investment due to resource availability, and contrary to theories of density dependence, which posit that increasing hatchling competition from later season clutches should drive selection for larger eggs. We provide empirical evidence that one species, *G. agassizii*, employs a bet‐hedging strategy that is a combination of two different bet‐hedging hypotheses. Additionally, we found some evidence for *G. morafkai* employing a conservative bet‐hedging strategy. (e.g., lack of intra‐ and interclutch variation in egg size relative to body size). Our novel adaptive hypothesis suggests the possibility that natural selection favors smaller offspring in late‐season clutches because they experience a more benign environment or less energetically challenging environmental conditions (i.e., winter) than early clutch progeny, that emerge under harsher and more energetically challenging environmental conditions (i.e., summer). We also discuss alternative hypotheses of sexually antagonistic selection, which arise from the trade‐offs of son versus daughter production that might have different optima depending on clutch order and variation in temperature‐dependent sex determination (TSD) among clutches. Resolution of these hypotheses will require long‐term data on fitness of sons versus daughters as a function of incubation environment, data as yet unavailable for any species with TSD.

## 1 Introduction

Scientists have long been interested in the trade‐off between offspring size and number in a reproductive bout within a population, and the fitness consequences for the female and her offspring associated with this trade‐off (Roff, 2002). However, empirical evidence for proposed theories and their associated hypotheses explaining the evolution of such a trade‐off are still debated, and are fueled by the lack of empirical support as well as contradictory evidence for each theory (Bernardo, 1996; Simons, 2011). In general, the energy available for reproduction is finite and stochastic depending on stored reserves and variable resource availability. Natural selection should favor strategies that allocate energy to the different components of reproductive output (i.e., offspring size and number) that maximize fitness of parents and their offspring. One component of such a strategy is offspring size (referred to as egg size from this point forward). Two basic theories attempt to explain how fitness is maximized via the trade‐off between egg size and number within and among clutches in a population: optimal egg size [OES] and bet‐hedging theory. Each of these theories has garnered support and challenges in the literature (Bernardo, 1996) that have led to numerous predictions (Table 1).

**Table 1:** Summary of reproductive strategies that have been offered to explain clutch and propagule size variation in a variety of organisms, with predictions for *Gopherus agassizii* (GOAG) and *Gopherus morafkai* (GOMO). MXREW is mean X‐radiograph egg width

Strategy	Predictions relative to egg size	Citation	*Gopherus* predictions
Optimal egg size	1. Mean egg size in a population is optimized in stable environments 2. Optimum size occurs when fitness advantage of a larger egg is equal to fitness disadvantage of producing fewer eggs 3. Clutch size varies more than egg size in a population	Smith and Fretwell (1974); Brockelman (1975)	Maximum egg width is constant across body sizes in a population (potentially above a minimum body size due to pelvic aperture constraints in smaller females) GOAG: egg width is constant across clutch number within years
Conservative bet‐hedging	Females produce eggs of uniform size, larger than long‐term optimum	Seger and Brockmann (1987); Philippi and Seger (1989)	No within‐clutch variation in egg width *and* no between‐year variation in egg width
Diversified bet‐hedging^a^	Females produce a range of egg phenotypes in each clutch drawn from a fixed distribution	Seger and Brockmann (1987); Philippi and Seger (1989)	Within‐clutch variation in egg width *and* between‐year variation in egg width
Dynamic bet‐hedging	When faced with unpredictable environments females increase intraclutch variation in egg size	Crean and Marshall (2009)	CV egg width and/or MXREW negatively correlated with precipitation
Nussbaum model of bet‐hedging	When resources are unpredictable late in the season, clutches will be smaller with larger eggs, resulting in interclutch variation in egg size	Nussbaum (1981)	GOAG: clutch size and number negatively correlated *and* MXREW and clutch number positively correlated GOMO: not applicable
Within‐generation bet‐hedging	One egg phenotype, but spatial and temporal spread of risk via placement of eggs	Hopper et al. (2003)	GOAG: observed production of multiple clutches within a season, oviposited at different locations GOMO: not applicable due to production of ≤1 clutch/reproductive bout
Sexual antagonistic selection and sex ratio	1. Males and females have different fitness optima in body size, which produces sexual size dimorphism 2. Adult body size is related to egg size 3. Females can adjust sex ratios to increase fitness, which create egg size variation within and among years	Trivers and Willard (1973); Trivers and Hare (1976)	Larger eggs are expected to produce male hatchlings, resulting in sexual size dimorphism with large body size in male tortoises that confers an advantage under male–male combat.

a Childs et al. (2010) argue that the adaptive coin‐flipping strategy (Cooper & Kaplan, 1982; Kaplan & Cooper, 1984) is the same as diversified bet‐hedging.

The main differences between OES and bet‐hedging theories are egg size variation (i.e., optimized vs. variable) and environmental condition (i.e., predictable vs. unpredictable) experienced by females within a population. Under OES theory, within a population, natural selection should optimize egg size while varying egg number within a reproductive bout (Brockelman, 1975; Smith & Fretwell, 1974). This theory is based on three main assumptions. (1) Larger eggs produce larger offspring that are assumed to be more fit (e.g., “bigger is better” hypothesis; Sinervo, 1990; Sinervo, Doughty, Huey, & Zamudio, 1992 but see Congdon et al., 1999). (2) Egg size should be independent of female size. However, see Congdon and Gibbons (1987) and Sinervo and Licht (1991) for a contrary “constraint” hypothesis, where the pelvic girdle might impose a limit on the passage of an optimal egg size. (3) Predictable environmental conditions enable optimization of egg size (Morrongiello, Bond, Crook, & Wong, 2012).

Although OES theory was formulated approximately 40 years ago, numerous examples challenge predictions arising from the theory (Bernardo, 1996; Roff, 2002). The predictions of OES theory are compromised in species that produce small clutch sizes (~1–10 eggs; Charnov & Downhower, 1995), and when there are architectural constraints on egg size (i.e., adaptive constraint hypothesis; Congdon & Gibbons, 1987; Sinervo & Licht, 1991; Rose & Judd, 1991; Forsman & Shine, 1995; Ljungström, Stjernstedt, Wapstra, & Olsson, 2016). More importantly, environments experienced by females and their offspring are typically more unpredictable than assumed under OES theory (Brockelman, 1975; Roff 2002; Morrongiello et al., 2012).

Under unpredictable environmental conditions, an optimal egg size in a single reproductive bout may not be advantageous under all possible environmental conditions and may increase the probability of total reproductive failure in a particular reproductive bout, thereby temporally increasing fitness variance while not necessarily reducing arithmetic mean fitness (Seger & Brockmann, 1987; Starrfelt & Kokko, 2012). In contrast, bet‐hedging theory is based on natural selection favoring any “strategy that reduces the temporal variance in fitness at the expense of lowered arithmetic mean fitness” (Ripa, Olofsson, & Jonzén, 2010). Thus, phenotypic diversification of egg sizes (Table 1) is a strategy to cope with environmental stochasticity in nature (Crean & Marshall, 2009; Hopper, Rosenheim, Prout, & Oppenheim, 2003; McGinley, Temme, & Geber, 1987) because provisioning eggs appropriately is more difficult in unpredictable environments (Einum & Fleming, 2004). Within populations, egg size diversification can occur among females within years or among years, among a female's reproductive bouts within a year, or within a female's discrete reproductive bout (Childs, Metcalf, & Rees, 2010).

Bet‐hedging theory has given rise to a number of competing hypotheses or predictions about egg size (Table 1). Although there are distinct differences among the various competing hypotheses, a combination strategy, where females use several bet‐hedging strategies, might be the “optimal bet‐hedging strategy,” thereby producing offspring variation within and among years (Olofsson, Ripa, & Jonzen, 2009). One hypothesis is the “conservative bet‐hedging” hypothesis. Females produce fewer, larger eggs, and each egg is larger than the long‐term optimal size, thereby ensuring that offspring are well provisioned in all environmental conditions, including poor conditions (Einum & Fleming, 2004; Philippi & Seger, 1989; Seger & Brockmann, 1987). Under the conservative bet‐hedging hypothesis, females are penalized in good years by investing more in larger eggs than necessary and forfeiting production of more eggs. However, this penalty is overcome during the lifespan of a long‐lived female by reducing fitness variance and increasing geometric mean fitness in an unpredictable environment.

As an alternative to conservative bet‐hedging, several hypotheses were developed to explain phenotypic diversification of egg sizes within a given year or reproductive bout: diversified bet‐hedging, where “egg sizes are drawn from a fixed distribution” (Olofsson et al., 2009; e.g., Crump, 1981; Seger & Brockmann, 1987) and dynamic bet‐hedging, where females “adaptively adjust” egg sizes within a reproductive bout based on environmental conditions (Crean & Marshall, 2009). Additionally, Nussbaum (1981) developed a bet‐hedging hypothesis to explain females producing smaller late‐season clutches with larger eggs as a strategy for the unpredictable environmental conditions during that time. Finally, among years, phenotypic diversification of egg sizes may best be explained via the adaptive coin‐flipping hypothesis (Cooper & Kaplan, 1982; Kaplan & Cooper, 1984). However, Childs et al. (2010) considers this hypothesis as another version of diversified bet‐hedging.

*Gopherus morafkai* (Cooper, 1861) and *G. agassizii* (Murphy et al., 2011) are closely related tortoise species (Murphy et al., 2011) that hybridize in a narrow contact zone in western Arizona (Edwards, Vaughn, et al., 2015). However, the reproductive strategies and environmental predictability for each species are very different, as are their ecology, morphology, and behavior (Table 2). *Gopherus morafkai* inhabits the eastern Sonoran Desert, a region that receives more predictable, and greater amounts of summer precipitation (monsoon rains) than *G. agassizii* habitat in the Mojave and western Sonoran deserts. In both species, abundance and quality of primarily annual plant food sources are critical for reproduction (e.g., clutch frequency, egg production, and clutch size) and are controlled by quantity and timing of winter/spring precipitation, which have known influences on the reproductive ecology of both species (Averill‐Murray, 2002; Henen, 1994, 1997; Lovich et al., 2015; Turner, Hayden, Burge, & Roberson, 1986). Although clutch sizes are similar between the two species, *G. agassizii* produces 1–3 clutches annually (Lovich et al., 2015), while *G. morafkai* ovulates a maximum of one clutch annually (Averill‐Murray et al. 2002, Averill‐Murray, Allison, & Smith, 2014). Female size usually explains very little variation in clutch size within populations of *Gopherus* (Averill‐Murray et al., 2014; and references cited therein). Across species of *Gopherus*, clutch size is not correlated with egg width, suggesting no trade off within the genus between number and size of eggs, but clutch size was correlated with female body size across populations of *G. polyphemus* (Averill‐Murray et al., 2014). Greater proportions of adult female *G. agassizii* reproduce each year than *G. morafkai* (Averill‐Murray et al., 2014). The completion of vitellogenesis also differs between the species (Table 2). *Gopherus agassizii* completes vitellogenesis prior to hibernation in the fall, while *G. morafkai* completes vitellogenesis after emergence from hibernation in the spring. Additionally, both species have temperature‐dependent sex determination (TSD). Clutches oviposited earlier in the reproductive season experience cooler nest temperatures and produce nearly all male hatchlings compared to clutches oviposited later (Baxter, Wilson, & Morafka, 2008), which leads to yet another hypothesis discussed below.

**Table 2:** Comparison of ecological characteristics between *Gopherus agassizii* and *Gopherus morafkai*

Trait	Species		Citation	Comments
	*Gopherus agassizii*	*Gopherus morafkai*		
Distribution	Mojave Desert and western Sonoran Desert	Central and eastern Sonoran Desert	Murphy et al. (2011)	Little is known about reproduction in *G. morafkai* in Mexico
Rainfall pattern in range	Dominated by winter rainfall in west, more biphasic (winter and summer) in the east	Strongly biphasic rainfall (winter and summer)	Turner (1982); Turner and Brown (1982)	Our study population of *G. agassizii* was in a winter‐dominated rainfall area
Predictability of rainfall	Low	High	Germano (1993)	Rainfall is a proxy for forage availability
Reproductive frequency	Up to 3 clutches/annum	Maximum 1 clutch/annum	Wallis et al. (1999); Averill‐Murray et al. (2014); Lovich et al. (2015)	
Major reproductive energy income strategy	Capital	Income	Henen (2004); Averill‐Murray (2002)	*G. agassizii* also uses “income” to produce later clutches
Completion of vitellogenesis	Prior to hibernation in the fall	After emergence from hibernation in the spring	Rostal et al. (1994); Averill‐Murray et al. (unpubl. data)	
Hatchling emergence	Fall emergence with rare overwintering	Fall emergence, with some potential overwintering	Luckenbach (1982); Averill‐Murray (2002)	Few data for *G. morafkai*
Food availability during hatchling emergence	Low	High	Morafka & Berry, 2002; Averill‐Murray et al. (2002)	

In summary, OES theory predicts that egg size should be independent of female size and that environmental predictability favors an optimal eggs size, while more challenging environments (especially for later clutches) should result in increased egg size in later compared to earlier clutches (Nussbaum, 1981). For example, *G. morafkai* may be more likely to exhibit an optimal egg size (e.g., less variability in egg size) in its relatively more predictable environment than that of *G. agassizii*. For bet‐hedging theories (i.e., diversified, and dynamic), selection favors an increase in within‐ and between‐clutch coefficients of variation in egg size produced by females. The constraint hypothesis does not obviate an “optimal egg size” per se, but posits the existence of constraints (e.g., the inside width of the pelvic girdle), which might limit the attainment of an OES in females below a certain size. Here we test several of these ideas, in particular those associated with the OES theory, a dynamic bet‐hedging hypothesis, and Nussbaum's (1981) hypothesis. We do this by analyzing patterns of variation in egg size in *G. agassizii* and *G. morafkai*. Finally, in TSD species like the ones we examine, female progeny may have different optima than male progeny (Roosenburg & Kelley, 1996), and thus, TSD affords an opportunity to have different OESs for the male and female progeny. This sexually antagonistic selection hypothesis (SASH) (Sinervo & Robart, 2016) is an extension of ideas developed by Trivers and Willard (1973) and Trivers and Hare (1976) with regard to the marginal gains of investment in sons versus daughters (Calsbeek & Sinervo, 2004).

We agree with Bernardo (1996) that none of the models and predications we describe in Table 1, and in subsequent tests in this paper, can fully explain the great variation observed in propagule size and number that exists in plants and animals. However, the models provide a heuristic framework to better understand the different solutions long‐lived organisms use to adapt to changing environmental conditions and resource availability when allocating resources to reproduction.

In this study, we collected reproductive data for *G. agassizii* and *G. morafkai* over a 16‐year period (1997‐2013) to investigate the evolution of egg size of these two desert species, one species in a less predictable and the other in a more predictable environment. More specifically, we tested predictions of OES and several hypotheses arising from bet‐hedging theory with our data (Table 1). In particular, we investigated whether mean egg width (i.e., a proxy for egg size) is (1) independent of female body size—a tenet of OES, (2) associated with short‐term precipitation variables, surrogates for environmental predictability and food availability—inconsistent with OES, (3) constrained by pelvic aperture sizes—a tenet of the constraint hypothesis, (4) associated with clutch size—contradictory to OES, and/or (5) associated with clutch order—a tenet of Nussbaum's hypothesis. Although testing for the existence of bet‐hedging with field collected data is difficult and requires revealing a reduced variance in fitness and the lowering of the arithmetic mean fitness, we investigated intraclutch egg width variation (i.e., a proxy for egg size variation), which will provide cues of the potential existence of bet‐hedging strategies in both species. We investigated whether intraclutch egg width variation (1) is associated with environmental predictability—a tenet of the dynamic bet‐hedging hypothesis, (2) varies within and among females—a tenet of the diversified bet‐hedging hypothesis but not for conservative bet‐hedging, and (3) varies among year—a tenet of the diversified bet‐hedging hypotheses but not for conservative.

In contrast to the predictions of OES and bet‐hedging, we find that the reproductive strategies in some species, such as tortoises, may actually favor a pattern of smaller egg size on later‐season clutches, if environmental conditions at that time are more benign than on early season clutches, contrary to the density‐dependent OES proposed by Brockelman (1975) and experimentally supported by Sinervo et al. (1992), Sinervo, Svensson, and Comendant (2000) and Nussbaum (1981). The pattern might also align with OES theory under SASH as discussed further below.

## 2 Materials and Methods

### 2.1 Study areas

Our two studies sites were located in the Desert Southwest of the United States. The *G. agassizii* site was located 13 km northwest of Palm Springs, California (33.951°N, 116.665°W), in the San Gorgonio Pass of the far western Sonoran Desert. Most rain is received in the winter, and summer rainfall is rare. Detailed descriptions of the study site are given in Lovich and Daniels (2000). The *G. morafkai* site was located in the Tonto National Forest near Sugarloaf Mountain, about 70 km northeast of Phoenix, Arizona (33.691°N, 111.509°W), in the northeastern Sonoran Desert. Here, biphasic rainfall (both winter and summer) is a common pattern. Detailed descriptions of the Sugarloaf Mountain site are given in Averill‐Murray (2002). Within our *G. agassizii* population, we collected egg width data for 8 years over a 16‐year period (1997–2013). For *G. morafkai*, we collected egg width data for nine consecutive years (1997–2005).

### 2.2 Species natural history

*Gopherus agassizii* and *G. morafkai* inhabit the Desert Southwest including portions of the Mojave and Sonoran deserts in the USA and Mexico. Both species are considered environmental engineers that excavate burrows used by a multitude of symbionts (Ernst & Lovich 2009). The distributions of these two species are geographically delineated by the Colorado River (Murphy et al., 2011) with few exceptions in a narrow hybrid zone (Edwards, Berry, et al., 2015). *Gopherus agassizii* generally inhabits valleys and alluvial fans of the Mojave and the western Sonoran deserts, while *G. morafkai* typically inhabits slopes, deep washes, and rocky hillsides within the eastern Sonoran Desert (Murphy et al., 2011). Both species produce small clutches of large eggs averaging about 4–5 eggs per clutch (Averill‐Murray et al., 2014; Lovich et al., 2015). The beginning of nesting typically occurs earlier in the year for *G. agassizii* (mid‐April; Ennen, Lovich, Meyer, Bjurlin, & Arundel, 2012; Lovich, Agha, et al., 2012) than *G. morafkai* (June; Averill‐Murray et al., 2014). Although *G. agassizii* occasionally constructs nest cavities outside of their burrows, clutches are more commonly deposited in nest cavities inside the burrows of both species (Averill‐Murray et al., 2014; Ennen et al., 2012). Incubation time and emergence differ depending on clutch order in *G. agassizii*. Hatchlings from first clutches emerge from the nest in August, while hatchlings from second and third clutches emerge in late September and October (Ennen et al., 2012). In *G. morafkai*, hatchlings generally emerge from the nest in late summer (i.e., September), although hatchlings of both species may overwinter in the nest (Averill‐Murray et al., 2014).

### 2.3 Data collection

At both sites, we used time‐area‐constrained searches to locate females (Crump & Scott, 1994; Walker, 2012), and we attached radio transmitters (Model R1540, Advanced Telemetry Systems, Inc., Isanti Minnesota or Wildlife Materials) to every adult female captured. We then located them every 3–10 days during April through early August for X‐radiography. X‐radiographs were collected using a Min‐Xray (HF‐80 or TR‐80, Northbrook, Illinois) with 3M Rare Earth (3M, St. Paul, Minnesota) or Custom X‐Ray Imaging Services (Phoenix, Arizona) cassettes, or a digital Canon X‐radiograph system (Melville, New York). We used Kodak, Imation, or Custom X‐Ray film. X‐radiograph exposures ranged from 60 to 65 kV for 0.08–0.24 s, depending on film or detector requirements.

Upon initial capture and for subsequent first annual relocation events, we recorded straight‐line carapace length along the medial axis (CL; measured in mm) for each individual. From the X‐radiographs, we measured X‐ray egg widths, clutch size (CS), clutch number (first clutch—CN1, second clutch—CN2, or third clutch—CN3), and X‐ray pelvic aperture width (here after pelvic aperture width). Widths of eggs and the inside of the pelvic aperture were measured (mm) directly from films with dial calipers or from digital images using the measurement tool in K‐PACS viewing software ( http://www.k-pacs.net/; accuracy ± 0.1 mm). This measurement likely overestimates pelvic aperture width by a small amount as it does not include surrounding soft tissue that is radio‐transparent. However, other than the study of Naimi, Znari, Lovich, Feddadi, and Ait Baamrane (2012) that included soft tissue, our measurement is consistent with other studies that examine morphological constraints on egg width in turtles (e.g., Congdon & Gibbons, 1987). We calculated mean X‐ray egg width (hereafter mean egg width) per clutch per female. We calculated the coefficient of variation (CV) for clutch size and intra‐ and interclutch egg width within and among clutches. Intraclutch CV was calculated within a clutch of eggs in both species. Interclutch CV was calculated from the mean egg widths among all clutches within each CN1, CN2, and CN3 for *G. agassizii* and among years for *G. morafkai*. However, our dispersion measurement is, in general, unreliable especially for the intraclutch calculations of smaller clutch sizes (e.g., 1, 2, and 3). Therefore, in our model selection analyses investigating CV of egg width within a clutch (intraclutch) in both species, we removed all clutches with less than four eggs. Our final sample size was 98 clutches for *G. agassizii* and 140 clutches for *G. morafkai* for our analyses.

We investigated the influence of the amount and timing (e.g., winter or summer) of precipitation on egg width and variability for several reasons. First, reproductive output, in theory (e.g., dynamic bet‐hedging or a plastic response), could be influenced by resource availability (e.g., plant productivity and water availability) for herbivores like tortoises, and secondly, reproductive output could be influenced by the female's response to unpredictability of the environment experienced by offspring after hatching. We collected precipitation totals from two biologically important periods (i.e., winter and summer) for each site. We collected winter precipitation (October–March) because germination of annual plants, which are staples in tortoise diets, in the Mojave and Sonoran deserts is associated with timing and quantity of winter precipitation (Beatley, 1974; Bowers, 2005; Hanson & Hanson, 2000). Both timing and amount of winter precipitation are associated with reproductive output in both species (Averill‐Murray, 2002; Henen, 1994, 1997; Lovich et al., 2015; Turner et al., 1986). We collected summer precipitation (i.e., June–September) as a variable associated with environmental conditions experienced by eggs and hatchlings (Averill‐Murray et al. 2002). Following the technique of Lovich, et al. (2014), we used WestMap ( http://www.cefa.dri.edu/Westmap/Westmap_home.php) to collect estimated short‐term precipitation variables from each study site. We estimated precipitation variables by calculating precipitation totals within each period and taking the average over a 3‐year period. The variables included mean winter precipitation of the prior 3 years (w.ppt) and mean summer precipitation of the current and prior 2 years (su.ppt). All precipitation values were collected in centimeters (cm).

### 2.4 Data analyses

Prior to conducting statistical analyses, we tested our data for normality in each species using a Shapiro–Wilks test. To improve linearity, reduce heteroscedasticity of variance, and facilitate comparisons to other studies (King, 2000), we used a log‐transformation for mean egg width, CL, CS, and pelvic aperture width, but not the precipitation variables. All analyses were conducted in R v. 3.3.2 (R Core Team 2016), and all log‐linear models included two random effects: year (referred to as YEAR) and individual tortoise (referred to as ID).

#### 2.4.1 OES predictions

We tested for influential predictors of mean egg width in both *G. agassizii* and *G. morafkai* using a multimodel selection approach. For each species, we used linear mixed‐effects models via the *lme4* package (Bates et al., 2015) fit with biologically relevant parameters. Models were compared using functions from the *MuMIn* package (Barton, 2016). The best model was selected by using Akaike' information criterion corrected for small sample size (AICc; Burnham & Anderson, 2002). We used all possible combinations (i.e., additively without interaction terms) of the following variables as fixed effects: CS, CN, CL, w.ppt, and su.ppt. CN was considered a categorical variable, and the inclusion of this fixed effect tested for interclutch variation in mean egg width. For *G. morafkai*, we excluded CN from our model selection because the species only ovulated one clutch annually. We excluded female *G. morafkai* that retained eggs over the winter (*N* = 5), which was an anomaly in the population. We used maximum‐likelihood estimation of parameters (rather than restricted maximum likelihood), which is appropriate for model comparisons with different fixed effects (Bates et al., 2015; Pinheiro & Bates, 2000). We identified the top model set as that including all models with a relative likelihood ≥0.05 (ΔAIC_c_ ≤* *6), excluding models from the candidate set if they were more complex versions of models having a lower AIC_c_ value (i.e., uninformative parameters; Arnold, 2010; Richards, Whittingham, & Stephens, 2011). Models were averaged, and average coefficient estimates and relative variable importance [RVI] were generated for fixed effects via functions from the *MuMIn* package (Barton, 2016). We report marginal *R* ^2^ ($R_{\left(m\right)}^{2}$) and conditional *R* ^2^ ($R_{\left(c\right)}^{2}$) for the global models (Nakagawa & Schielzeth, 2013).

We investigated the presence of an architectural constraint of the pelvic aperture by conducting analyses of covariance (ANCOVA) to test the homogeneity of slopes between the relationships of pelvic aperture width and mean egg width with CL (see Congdon & Gibbons, 1987). If slopes are parallel and the largest egg width (x‐radiographed egg width) is larger than the smallest pelvic aperture width in the populations, we concluded that there is a constraint on egg size by the pelvic aperture (Iverson & Smith, 1993; Lovich, Madrak, et al., 2012). In these analyses, we used full‐factorial, mixed‐effects regression models via functions from the *lme4* package (Bates et al., 2015) with individual tortoise and year as random effects. Because *G. agassizii* produces multiple clutches per annum, we included CN as an additive effect in the analysis. We considered an effect significant (α = 0.05) if the 95% confidence intervals (CI) did not overlap with zero.

#### 2.4.2 Bet‐hedging predictions

We tested for influential predictors of intraclutch egg width variation using the same multimodel approach as that for the OES investigation, instead using CV of egg width as the response variable. In addition, to further understand intra‐ and interclutch egg size variation, we conducted restricted likelihood ratio tests (RLRT) using 1,000 simulations via a function from the *RLRsim* package (Scheipl, Greven, & Kuechenhoff, 2008). These RLRTs were conducted on linear mixed‐effects models in both species. We conducted a RLRT on models predicting intraclutch egg width variation and mean egg width using CN and CL as fixed effects for *G. agassizii*, but only used CL as a fixed effect in our *G. morafkai* model and subsequent RLRT.

## 3 Results

We measured 608 eggs from 140 clutches of 20 *Gopherus agassizii* females and 350 eggs of 66 clutches from 19 *G. morafkai* females (Table 3). In general, clutch size varied more than egg width based on CV in both species, and clutch size was more variable for *G. agassizii* when compared to *G. morafkai* (Table 3). In addition, *G. morafkai* produced larger mean clutch sizes than *G. agassizii*; however, *G. agassizii* produced larger eggs and more clutches per year than *G. morafkai* (Table 3). Both species exhibited greater interclutch variation in egg width than intraclutch variation (Table 3). Interclutch CV was significantly larger in CN1 (one‐way analysis of variance, *F*_(1,15)_ = 37.45, *p *<* *.001) and CN2 (*F*_(1,15)_ = 6.39, *p *=* *.02) for *G. agassizii* when compared to *G. morafkai,* while there was no difference in intraclutch CV between the species (Table 3).

**Table 3:** Summary statistics of clutch size and egg width (mm) by clutch order for *Gopherus agassizii* and *Gopherus morafkai*. N^1^ and N^2^ represent the number of unique clutches and the number of eggs measured, respectively. CV^1^ represents interclutch variation as measured by the coefficient of variation, while CV^2^ presents intraclutch variation measured by the coefficient of variation. *SE* represents standard error

Species	Clutch				Egg width				
	N^1^	Order	Mean Size (*SE*)	CV	N^2^	Mean (*SE*)	Min, Max	CV^1^	CV^2^
*Gopherus agassizii*	75	1	4.21 (0.17)	0.36	316	38.96 (0.32)	30.99, 45.0	0.07	0.03
	56	2	4.70 (0.22)	0.35	263	38.98 (0.29)	33.0, 44.4	0.06	0.03
	9	3	3.22 (0.43)	0.40	29	37.30 (0.95)	31.0, 40.9	0.08	0.02
*Gopherus morafkai*	66	1	5.30 (0.20)	0.31	350	38.00 (0.19)	33.6, 44.7	0.04	0.03

### 3.1 Optimal egg size

#### 3.1.1 Mean egg width

Our model selection revealed different influential predictors for mean egg width in each species (Table 4). Excluding models containing uninformative parameters from the results, the top model set included a single model (CL + CN) predicting mean egg width for *G. agassizii*. The coefficient estimate for CL was positively associated (0.779 ± 0.156 *SE*) with mean egg width. Mean egg width of CN2 (–0.009 ± 0.005 *SE*) and CN3 (–0.044 ± 0.0.010) was smaller relative to CN1, indicating that mean egg width decreased with clutch order. CS and winter/summer rainfall were unimportant in predicting mean egg width. The data fit the global model reasonably well, with $R_{\left(m\right)}^{2}$ = 0.394 and $R_{\left(c\right)}^{2}$ = 0.870.

**Table 4:** The top five models and the null model from the AICc model selection predicting mean egg width for *Gopherus agassizii* and *Gopherus morafkai*. These linear mixed models used year and individual tortoises as random effects. The top model set for each species is indicated by *. Abbreviations are as follows: CL (carapace length), CN (clutch number), CS (clutch size), w.ppt (mean winter precipitation of the prior 3 years), and su.ppt (mean summer precipitation of the current and prior 2 years)

Species/Model	df	AICc	δ	Weight
*Gopherus agassizii*				
*CL + CN	7	−550.2	0.0	0.294
CL + CN + w.ppt	8	−549.6	0.6	0.213
CL + CN + CS	8	−548.5	1.7	0.128
CL + CN + su.ppt	8	−548.5	1.7	0.126
CL + CN + CS + w.ppt	9	−547.8	2.4	0.091
NULL	4	−518.7	31.5	<0.0001
*Gopherus morafkai*				
*CL + su.ppt + w.ppt	7	−275.7	0	0.196
*CL + w.ppt	6	−275.6	0.1	0.186
*CL	5	−275.3	0.4	0.160
CL + su.ppt	6	−275.3	0.4	0.157
CL + CS + w.ppt	7	−274.0	1.7	0.081
NULL	4	−266.1	9.6	0.001

In *G. morafkai*, the top model set included three models with influential variables for predicting mean egg width (Table 4). Model averaging of these three top models found only CL (0.454 ± 0.121 *SE*; RVI = 1.0; *p* < .001) to be a significant predictor of mean egg width, while winter (0.001 ± 0.001 *SE*; RVI = 0.70; *p* = .542) and summer (−0.001 ± 0.002 *SE*; RVI = 0.36; *p* = .291) precipitation were unimportant (CI overlapping zero). As with the *G. agassizii* analysis, the data fit the global model reasonably well, with $R_{\left(m\right)}^{2}$ = 0.392 and $R_{\left(c\right)}^{2}$ = 0.801.

**Figure 1:**
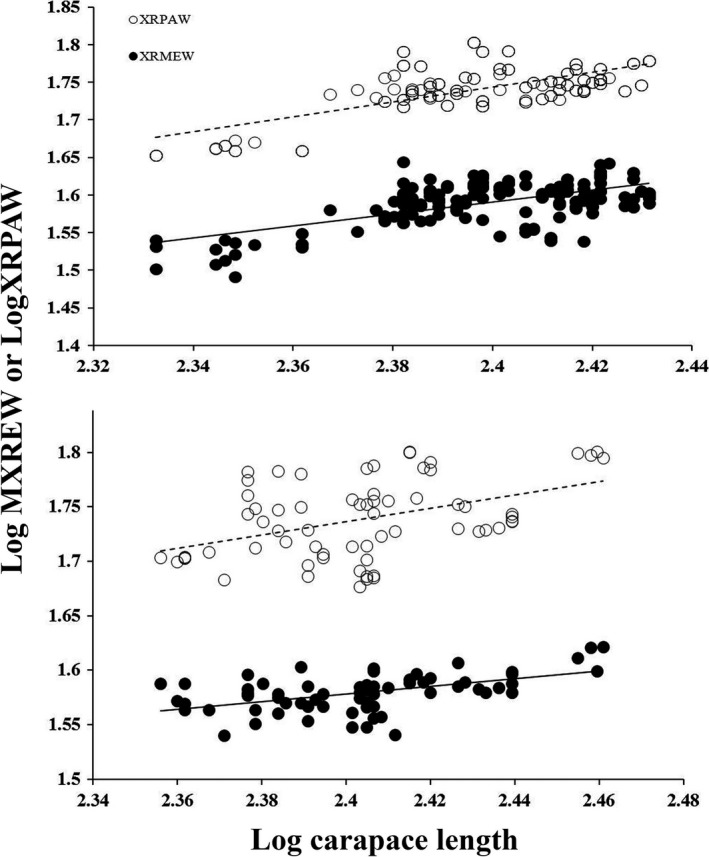
The relationship between X‐radiograph pelvic aperture width (XRPAW) and mean egg width (XRMEW) with female body size (carapace length) for a *Gopherus agassizii* population in Southern California (above) and a *Gopherus morafkai* population in south‐central Arizona (below)

For *G. agassizii*, the slopes of mean egg width and pelvic aperture width did differ across CL (interaction term, CI = 0.042; 0.341; Figure 1), and the largest mean egg width (44.1 mm) was less than the smallest pelvic aperture width (45.0 mm). Both of these results suggest no morphological constraint on egg size exists for *G. agassizii*. CL (CI = 0.400; 1.015) and CN3 (CI = −0.042; −0.006) were significant in our *G. agassizii* analysis. For *G. morafkai*, pelvic aperture width increased more steeply relative to CL than did mean egg width (interaction term, CI = 0.019; 0.501; Figure 1), suggesting that egg width was not constrained by the pelvic aperture. Mean egg width increased with CL in our *G. morafkai* analysis (CI = 0.022; 0.727).

### 3.2 Bet‐hedging and variation in egg widths

Our model selection revealed different important predictors for intraclutch egg width variation in each species (Table 5). The top model set for *G. agassizii* included two models (CS + su.ppt & CS). Model averaging of these two models found only clutch size (0.011 ± 0.005 *SE*; RVI = 1.0; *p* = .042) to be a significant predictor of intraclutch egg width variation. The data fit the global model poorly, with $R_{\left(m\right)}^{2}$ = 0.096 and $R_{\left(c\right)}^{2}$ = 0.314; therefore, the explanatory variable of CS carried very little predictive power in the global model results. In *G. morafkai*, the null model (i.e., no effects included) was the top model predicting intraclutch egg width variation (Table 6), and the data fit the global model poorly, with $R_{\left(m\right)}^{2}$ = 0.074 and $R_{\left(c\right)}^{2}$ = 0.289.

**Table 5:** The top models and the null model from the AICc model selection predicting intraclutch egg width variation (i.e., coefficient of variation) for *Gopherus agassizii* and *Gopherus morafkai*. These linear mixed models used year and individual tortoises as random effects. The top model set for each species is indicated by *. Abbreviations are as follows: CL (carapace length), CS (clutch size), w.ppt (mean winter precipitation of the prior 3 years), and su.ppt (mean summer precipitation of the current and prior 2 years). CN (clutch number) did not appear in the top models

Species/Model	df	AICc	δ	Weight
*Gopherus agassizii*				
*CS + su.ppt	6	−588.2	0	0.164
*CS	5	−587.9	0.29	0.141
CS + w.ppt	6	−586.3	1.86	0.065
CL + CS + su.ppt	7	−586.1	2.05	0.059
NULL	4	−586.1	2.05	0.059
su.ppt	5	−586.1	2.06	0.058
*Gopherus morafkai*				
NULL	4	−352.3	0	0.204
CS	5	−351.4	0.82	0.136
w.ppt	5	−351.3	0.92	0.129
CL	5	−350.5	1.75	0.085
CS + w.ppt	6	−350.4	1.82	0.082
su.ppt	5	−349.9	2.4	0.062

**Table 6:** The variance, standard error of the variance, and the results of the restricted likelihood ratio test for the random effects of individual (ID) and year (YEAR) in our linear mixed models predicting mean egg width and intraclutch egg width variation (i.e., coefficient of variation) in two desert tortoise species

Parameter	Variance	*SE*	RLRT	Pr(>|z|)
Mean egg width				
*Gopherus agassizii*				
ID	0.0024	0.049	141.680	<0.0001
YEAR	0.0001	0.009	3.918	0.01680
Residuals	0.0006	0.025		
*Gopherus morafkai*				
ID	0.0009	0.0292	29.556	<0.0001
YEAR	0.0001	0.0078	1.280	0.1098
Residuals	0.0004	0.0204		
Intraclutch egg width				
*Gopherus agassizii*				
ID	0.00004	0.006	7.213	0.002
YEAR	0.0000	0.000	0.000	0.420
Residuals	0.0001	0.011		
*Gopherus morafkai*				
ID	0.00002	0.004492	1.5767	0.0939
YEAR	0.0000	0.00000	0.00000	1.000
Residuals	0.0001	0.009539		

Results from the RLRTs differed between species and random effect depending on the response variable (Table 6). In *G. agassizii*, the random effects of ID and YEAR both explained a significant portion of the variance in mean egg width. Mean egg widths are more consistent among years in *G. morafkai*, with only ID explaining a significant portion of the variation. ID explained a significant portion of the variance in intraclutch egg width model for *G. agassizii*, but not for *G. morafkai*; while the random effect of YEAR was not significant in either species (Table 6). In the global model predicting intraclutch egg width variation, the random effect of YEAR explained none (zero) of the variance.

## 4 Discussion

Mean egg width was less variable than mean clutch size in both species, and mean clutch size and mean egg width were less variable in *G. morafkai* than *G. agassizii*, as might be expected under OES theory in the relatively more predictable eastern Sonoran Desert environment. However, neither species conformed to other predictions of OES theory. First, we observed a positive relationship between mean egg width and maternal body size in both species, a phenomenon not unusual in other turtle species (Ryan & Lindeman, 2007). Second, the relationship between mean egg width and clutch order in *G. agassizii* was the inverse of what would be predicted. Under theories of density competition of OES (Brockelman, 1975), offspring size should increase in later clutches when density‐dependent competition will be predictably more intense. For example, experiments confirm an advantage to the production of larger second‐clutch eggs in the side‐blotched lizard (Sinervo et al., 1992, 2000). *Gopherus agassizii* also exhibited phenotypic diversification of mean egg width among clutches and among females within a given year, and egg width also varied among years. Finally, intraclutch egg width variation differed among *G. agassizii* females.

We found varying support for the different bet‐hedging hypotheses. The lack of a relationship between precipitation and intraclutch egg width variation does not support dynamic bet‐hedging hypothesis in either species (Crean & Marshall, 2009). *Gopherus morafkai* exhibited no variation between individual females or years in egg width, suggesting that females produce a single egg size phenotype relative to individual body size. Turtle species tend to apply bet‐hedging strategies in less predictable environments, where individuals demonstrate variable or more frequent reproductive output than individuals in other species inhabiting more predictable environments (Iverson, 1992). For example, less frequent reproductive output in *G. morafkai* (i.e., females often skipping reproduction in a year) is associated with high predictability of rainfall in the Sonoran Desert (Averill‐Murray et al., 2014). Even though their environment may be more predictable relative to that of *G. agassizii*,* G. morafkai* hatchlings still face harsh desert conditions, so when females do reproduce they appear to apply a conservative bet‐hedging strategy by producing eggs of consistent size between clutches (i.e., little intraclutch variation in egg size).

*Gopherus agassizii* exhibited significant variation among females but not among years in intraclutch egg size variation, and this pattern would provide potential support for the diversified bet‐hedging hypothesis. Although bet‐hedging strategies are best evaluated by examining within‐ and between‐clutch CVs, we caution the reader about our results using these measurements. Our global models of intraclutch egg width variation explained very little variance (<10% for the marginal *R* ^2^), likely due the low clutch sizes (~mean of five eggs) and resultant unreliable estimates of standard deviation for egg width within both species. Although our model selection predicting intraclutch egg width variation found CS an important, positive variable, this explanatory variable of clutch size carried very little predictive power in the global model results. We evaluate support for other bet‐hedging strategies, in particular Nussbaum (1981) and within‐generation bet‐hedging, based on mean egg width more thoroughly below.

Our study population of *G. agassizii* exhibits a within‐generation bet‐hedging strategy (Hopper et al., 2003; Root & Kareiva, 1984) by producing multiple clutches within a year and spreading risk of reproductive failure spatially (females oviposit each clutch at different location) and temporally (over 4 months) (see Lovich, Yackulic, et al., 2014; Lovich, Ennen, et al., 2015). In addition, the study *G. agassizii* population appears to utilize an additional strategy to combat the unpredictable nature of the western Sonoran Desert and Mojave Desert. Conditions in that region became increasingly arid throughout the Pleistocene, especially with the loss of the summer monsoon in the middle Holocene Epoch (see Morafka & Berry, 2002). In *G. agassizii*, an inverse relationship between mean egg size and clutch order within a reproductive season exists, which is opposite of the bet‐hedging hypothesis developed by Nussbaum (1981), and theories of density‐dependent OES (discussed above). Under Nussbaum's hypothesis, late‐season clutches have fewer eggs to hedge against an increased probability of failure (i.e., first clutch 5% and second clutch 38% failure) but eggs are larger due to the fractional egg size theory (Ricklefs, 1968). The Nussbaum bet‐hedging hypothesis is supported by data for another turtle species, *Carettochelys insculpta* (Doody, George, & Young, 2003).

### 4.1 Species comparison

Differences in the evolution of reproductive strategies, including egg size, can be explained in that the two species we studied occupy very different climate spaces and experience differences in environmental variation. *Gopherus agassizii* experiences the lowest amount, greatest seasonality, and the greatest variation of annual precipitation among North American tortoises, while precipitation within the range of *G. morafkai* is about 80% greater and more predictable than that of experienced in the range of *G. agassizii* (Germano, 1993). *Gopherus morafkai* females, in their relatively predictable environment, allocate energy acquired from spring‐germinated plants immediately prior to the reproduction season (“income energy”; Drent & Daan, 1980; Henen, 2004) to egg production. In contrast, *G. agassizii* females cope with greater environmental uncertainty by increasing their body energy content, undergo vitellogenesis before winter, and use reserves (“capital energy”; Drent & Daan, 1980) the following spring to produce eggs within the first clutch. They also acquire energy by foraging during spring when resources are available, which can contribute to production of subsequent clutches during the reproductive season (Henen, 1997; Lovich et al., 2015).

Precipitation did not affect egg size in either species potentially for two reasons. First, neither winter nor early spring precipitation in the seasons immediately prior to oviposition contributed to egg size in *G. morafkai* (Averill‐Murray, 2002) or contributed to annual egg production, clutch frequency, or the percentage of reproductive females in *G. agassizii* (Lovich et al., 2015), except for the latter two variables during an exceptionally strong El Niño event (Lovich et al., 2015). Alternatively, with only 7–9 years of reproductive data, our study may have lacked the statistical power to recover interannual egg size differences related to the range of precipitation values we observed. This is especially true given the nonlinear relationship between precipitation quantity and biomass production of annual food plants for tortoises (Lovich et al., 2015).

### 4.2 Inverse relationship explanation

The inverse relationship between egg size and clutch order in our population could be explained by three hypotheses: resource constraint or plastic response, benign environment, or the sexually antagonistic selection hypothesis (SASH). In the resource constraint (i.e., plastic response) hypothesis, the reproductive cycle of *G. agassizii* may explain the inverse relationship as constraints arising from capital breeding. In turtles, ovarian follicles for first, second, and third clutches are ovulated over several months (Moll, 1979), and clutches of *G. agassizii* are formed and oviposited in a like manner over a given year with second and third clutches of shelled eggs forming from May to mid‐July and June to late July, respectively (Lovich, Agha, et al., 2012). Levels of yolk‐ and shell‐forming compounds circulating in the blood stream are depleted to their lowest level in June (Lance & Rostal, 2002; Lance et al., 2002). As a result, second and particularly third clutches may not have enough yolk‐ or shell‐forming compounds to produce eggs comparable in size to those of eggs in the first clutch, which are provisioned from resources harvested during the prior year (Henen, 1997, 2004; Rostal, Lance, Grumbles, & Alberts, 1994). In another multiclutch per annum species, *Kinosternon subrubrum*, females produced smaller eggs in late‐season clutches potentially due to the depletion of body fat reserves later in the reproductive season (Wilkinson & Gibbons, 2005). However, the resource constraint hypothesis has not found universal support in reptile species that produce multiple clutches in a reproductive season (see Doody et al., 2003; Nussbaum, 1981).

Alternatively, seasonal changes, especially in precipitation, that affect the posthatching environment may provide an evolutionary basis for why *G. agassizii* produces smaller eggs in their third clutch. Hatchlings of late‐season clutches emerging during a period of relatively benign environmental conditions (e.g., cooler temperatures) may need less maternal investment relative to hatchlings of earlier clutches (i.e., benign environment hypothesis). For example, third clutches of shelled eggs in *G. agassizii* were visible in X‐radiographs between 16 June and 28 July (Lovich, Agha, et al., 2012), which pushes emergence dates for hatchlings conservatively into October and November, a period of less extreme temperatures, the onset of the rainy season, and only 1 or 2 months away from germination of annual food plants in December that are at accessible heights for neonates to utilize (Morafka & Berry, 2002). Neonates and juveniles may be active and forage during the winter (Wilson, Morafka, Tracy, & Nagy, 1999). Therefore, natural selection potentially favors lesser‐provisioned eggs in the third clutch relative to earlier clutches of eggs (i.e., first and second) because offspring would emerge with enough energy to persist into December when forage is more likely to be ample, unlike hatchlings that emerged from first and second clutches in summer months when summer precipitation and forage is sparse. Interestingly, third‐clutch eggs of *G. agassizii* were statistically similar with *G. morafkai* eggs (mixed model: *F*_(1,75.0)_ = 0.005, *p *=* *.94) after removing the effect of maternal size. The fact that egg sizes are not significantly different between the species and, therefore, are likely provisioned similarly suggests that third‐clutch eggs probably emerge at a point of somewhat more predictable forage like *G. morafkai* hatchlings in the northeastern Sonoran Desert, where summer precipitation predictably triggers germination of food plants for hatchlings (Averill‐Murray, 2002). For example, Averill‐Murray et al. (2014) states, “Investment by *G. morafkai* of its entire annual reproductive output in a single clutch of relatively small eggs suggests that a more productive posthatching environment during the typical monsoon season may contribute to higher average juvenile survival than for *G. agassizii*.” However, the egg size differences among clutches might not necessarily create a measurable fitness difference among the hatchlings of the various clutches, and hatchling fitness among clutches (c.f., Sinervo et al., 1992) should be investigated to address this hypothesis.

Another explanation for the inverse relationship is related to SASH and maternal adjustment of sex ratio within and among reproductive bouts. In numerous species of reptiles, progeny gender is under environmental influence, especially temperature (Harlow & Taylor 2000, Elf et al. 2002, Milnes et al. 2002, Shine et al. 2002), and is thought to have an adaptive explanation (Shine 1999). Given that the female is responsible for the nest location, females can control the sex ratio of clutches by varying the depth and/or location where eggs are buried in nests (Packard et al. 1987, Roosenburg 1996; Morjan & Janzen 2003; Baxter et al., 2008). For example, Roosenburg (1996) speculates that female diamond‐backed terrapin (*Malaclemys terrapin*) nest site selection should be plastic depending on the size of a female's eggs. He suggested that females that lay small eggs should oviposit in places where those eggs will develop into males in the diamond‐backed terrapin potentially because there is not a significant premium on male size that might arise for male–male contests. In contrast, if a mother is going to produce a clutch with very large eggs, then she should lay those eggs in a warm place, where they will develop into female because there is a significant premium on larger females producing more offspring.

Here we suggest that the egg size premium placed on large size in *Gopherus* males, which exhibit male–male combat rituals, should favor large eggs and an earlier oviposition date for male‐biased clutches. Ewert, Jackson, and Nelson (1994) suggested that patterns of sex determination in turtles are related to future growth potential and maturation, factors that affect sexual sized dimorphism. Baxter et al. (2008) demonstrated that *G. agassizii* clutches oviposited early in the reproductive season produced almost all male hatchlings, while late‐season clutches produced only females. Given that total investment is constrained, Trivers (1972) and in related sex ratio theory, Trivers and Hare (1976) and Trivers and Willard (1973), suggest that females should invest in the sex from which the marginal gains in fitness are the greatest. Thus, if *Gopherus* females in good condition can produce relatively large eggs, or females laying earlier in the season can produce large eggs, they should produce male‐biased clutches. Conversely, females in poor condition or later‐season clutches, which might have smaller eggs (due to the physiological constraint, discussed above), should produce females offspring (with less of a fitness cost than if they produced males). Accordingly, the shift in egg size we observe might be a case of OES subject to SASH (Sinervo & Robart, 2016). This also requires females to adjust sex ratio of their clutch by burying them at the appropriate depth such that nest temperatures will generate the predicted sex ratio, a behavior that may be used by *G. agassizii* (Ennen et al., 2012).

## 5 Conclusions

No single reproductive strategy can explain the full range of variation observed in egg and clutch size of an organism. As noted by Bernardo (1996), maternal and offspring resource environments are often far‐removed in time and space, “…making it difficult to state with generality how resource availability affects [per offspring investment].” In this study, we report several findings related to theories of maternal investment. First, we provide empirical evidence of a species expressing a bet‐hedging strategy that is a combination of multiple bet‐hedging hypotheses that ultimately support previous simulation results by Olofsson et al. (2009). In this case, *G. agassizii* exhibited within‐generation bet‐hedging strategy (i.e., spreading risk temporally and spatially) and another strategy, where egg phenotype diversification occurs among clutches within a reproductive season and mean egg size is inversely related to clutch order. Second, we posit a novel bet‐hedging hypothesis (i.e., benign environment hypothesis) explaining the inverse relationship between egg size and clutch order. Natural selection could favor smaller late‐clutch offspring because they emerge in a more benign environment. Next, our benign environment hypothesis is contrary to theories of density dependence, which posit that increasing competition among offspring of later‐season clutches should drive selection for larger eggs on later clutches. Finally, the inverse relationship between egg size and clutch order might be explained by SASH and females adaptively adjusting sex ratios.

## Conflict of Interest

None declared.

## Author Contributions

All authors contributed to the design, acquisition, analyses, or interpretation of the data, and all authors either drafted or critically revised the manuscript. All authors have approved of the final version of this manuscript and agree to be accountable for the content's accuracy.

## Data Accessibility

The data associated with this manuscript are available from: https://dx.doi.org/10.5066/F7JS9NN9. These data are published following U.S. Geological Survey data policies: Lovich, J.E., Ennen, J.R., Averill‐Murray, R.C., and Agha, M., 2017, Desert Tortoise Reproductive Ecology and Precipitation, Mojave and Sonoran Deserts—Data: U.S. Geological Survey data release, https://dx.doi.org/10.5066/F7JS9NN9.
